# Nitric Oxide Impacts Human Gut Microbiota Diversity and Functionalities

**DOI:** 10.1128/mSystems.00558-21

**Published:** 2021-09-14

**Authors:** Marion Leclerc, Cassandre Bedu-Ferrari, Lucie Etienne-Mesmin, Mahendra Mariadassou, Lucie Lebreuilly, Seav-Ly Tran, Laurence Brazeau, Camille Mayeur, Julien Delmas, Olivier Rué, Sylvain Denis, Stéphanie Blanquet-Diot, Nalini Ramarao

**Affiliations:** a Université Paris-Saclay, INRAE, MICALIS Institute, Jouy-en-Josas, France; b Université Clermont Auvergne, INRAE, UMR 454 MEDIS, Clermont-Ferrand, France; c Université Paris-Saclay, INRAE, MaIAGE, Jouy-en-Josas, France; d Université Clermont Auvergne, Inserm U1071, M2iSH, USC-INRA 2018, Clermont-Ferrand, France; e CHU Clermont-Ferrand, Laboratoire de Bactériologie, Centre de Biologie, Clermont-Ferrand, France; f Université Paris-Saclay, INRAE, BioinfOmics, MIGALE Bioinformatics, Jouy-en-Josas, France; Duke University School of Medicine

**Keywords:** nitric oxide, microbiota, anaerobic digester, single strain, diversity, metabolomics, functional analysis

## Abstract

The disruption of gut microbiota homeostasis has been associated with numerous diseases and with a disproportionate inflammatory response, including overproduction of nitric oxide (NO) in the intestinal lumen. However, the influence of NO on the human gut microbiota has not been well characterized yet. We used *in vitro* fermentation systems inoculated with human fecal samples to monitor the effect of repetitive NO pulses on the gut microbiota. NO exposure increased the redox potential and modified the fermentation profile and gas production. The overall metabolome was modified, reflecting less strict anaerobic conditions and shifts in amino acid and nitrogen metabolism. NO exposure led to a microbial shift in diversity with a decrease in Clostridium leptum group and Faecalibacterium prausnitzii biomass and an increased abundance of the *Dialister* genus. Escherichia coli, Enterococcus faecalis, and Proteus mirabilis operational taxonomic unit abundance increased, and strains from those species isolated after NO stress showed resistance to high NO concentrations. As a whole, NO quickly changed microbial fermentations, functions, and composition in a pulse- and dose-dependent manner. NO could shift, over time, the trophic chain to conditions that are unfavorable for strict anaerobic microbial processes, implying that a prolonged or uncontrolled inflammation has detrimental and irreversible consequences on the human microbiome.

**IMPORTANCE** Gut microbiota dysbiosis has been associated with inflammatory diseases. The human inflammatory response leads to an overproduction of nitric oxide (NO) in the gut. However, so far, the influence of NO on the human gut microbiota has not been characterized. In this study, we used *in vitro* fermentation systems with human fecal samples to understand the effect of NO on the microbiota: NO modified the microbial composition and its functionality. High NO concentration depleted the microbiota of beneficial butyrate-producing species and favored potentially deleterious species (E. coli, E. faecalis, and P. mirabilis), which we showed can sustain high NO concentrations. Our work shows that NO may participate in the vicious circle of inflammation, leading to detrimental and irreversible consequences on human health.

## INTRODUCTION

Gut microbial communities stably coexist in the gastrointestinal tract as a structural unit forming a complex anaerobic trophic network, ensuring the conversion of dietary nutrients into a wide variety of metabolites (1). These communities generate energy supply during fermentation metabolism, mainly from polysaccharides, further transformed into short-chain fatty acids (SCFAs), such as acetate, propionate, and butyrate, and gases (H_2_, CO_2_, N_2_, and CH_4_ for methane-producing individuals). Gut microbiota can also generate energy from nutrients in the anaerobic intestinal environment through ethanolic or lactic fermentations.

Disruptions of intestinal homeostasis have been associated with a wide range of pathological states, including inflammatory bowel disease (IBD), colorectal cancer (CRC), and metabolic disorders, such as obesity and type 2 diabetes (2–4). These alterations in the intestinal environment trigger lower microbial resilience and higher susceptibility to pathogens or pathobiont emergence and are commonly associated with aberrant inflammatory responses (5).

Among the host mediators of intestinal inflammation, nitric oxide (NO) is an essential component of host immunity. NO plays an important role during infections by limiting microbial proliferation (6). Known to be highly reactive, this signaling molecule can target nucleic acids and lipid components with bactericidal effects. Macrophages mainly localized at the vicinity of the intestinal barrier, and epithelial cells can release NO in the intestinal crypts that diffuses toward the luminal content. Very few studies have estimated the concentration of NO *in vivo*. Under physiological conditions, the NO basal concentration allows to control commensal microbial populations and maintain the integrity of the intestinal epithelial barrier (7). In contrast, during inflammation, NO is released as pulses, and a study based on chemiluminescence technique in patients suffering from ulcerative colitis revealed that NO concentrations would be more than 100 times higher in inflamed colonic luminal content than in healthy individuals (8). A predictive mathematical model simulating NO diffusion and reaction in colonic crypts determined the NO concentration to reach about 0.2 μM (9). However, a precise measure of NO production and impact during inflammation is still missing.

Studies on pathogenic bacteria showed that bacteria are differently sensitive to NO. In some cases, NO is a potent antimicrobial agent (6). However, some pathogenic bacteria can detect NO via dedicated sensor proteins and switch on the expression of detoxifying enzymes to resist NO-induced damage (10, 11). Others, such as pathogenic Escherichia coli, stimulate NO production by enterocytes through bacterial stimuli in order to decrease intestinal barrier permeability and invade host tissue (12). Salmonella are NO resistant and gain a competitive advantage over the gut microbiota under high NO conditions (13). It is noteworthy that some lactobacilli protect the integrity of the intestinal barrier by reducing excessive NO production (14).

Thus, NO is both an efficient antimicrobial host defense mechanism and a maintainer of homeostasis in the gut microbiota. However, although disproportionate inflammatory responses have been associated with the development and the progression of gut inflammatory diseases (15), the impact of NO during inflammation on the gut microbiota is unknown. NO, as a mediator of intestinal inflammation, could modulate the ecological structure of the gut microbial communities and directly favor bacterial populations able to use nitrosative products as substrates/energy sources or lead to stress response by sensitive bacterial groups.

In this study, we highlight the effects of NO on the human gut microbiota. *In vitro* fermentation systems inoculated with human fecal microbiota were used to monitor and analyze the impacts of repetitive NO pulses on fermentation parameters (redox potential and gas production), microbial composition, and metabolomics.

## RESULTS

### NO modifies human gut ecosystem fermentation characteristics.

**FIG 1:**
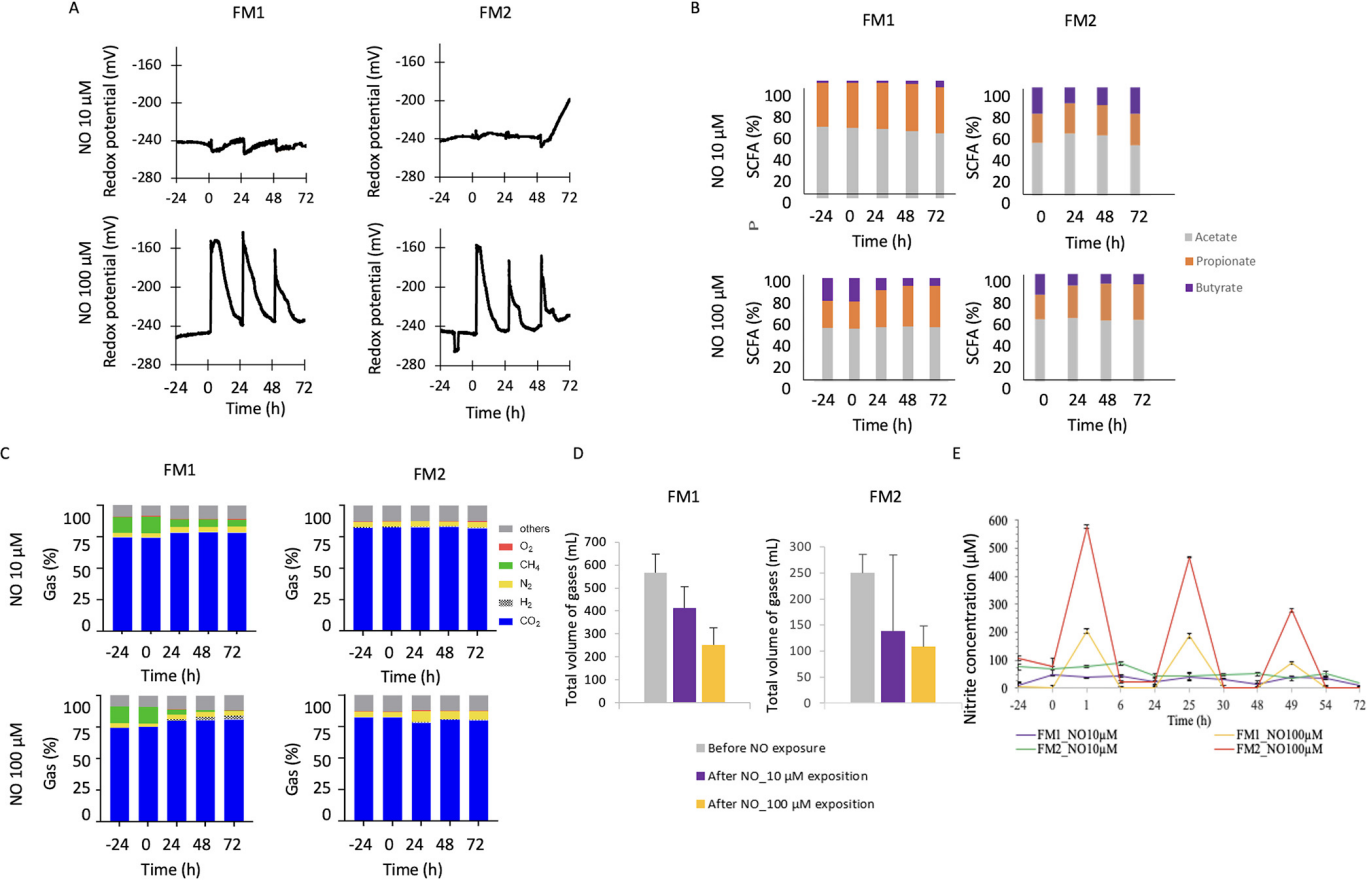
NO impact on the human gut ecosystem fermentation activities. The impact of 10 μM and 100 μM NO administered in 3 pulses on two fecal microbiota (FM1 and FM2) was measured in terms of redox potential (A), SCFA production (B), gas (O_2_, CH_4_, N_2_, CO_2_, H_2_) proportions (C), and collected gas volume (D). NO concentration was indirectly assessed through the measurement of total accumulated nitrite by the Griess reagent system (E).

Following a stabilization phase of 24 h, three pulses of NO (at 0, 24, and 48 h) at 10 μM and 100 μM were applied to two healthy subject fecal microbiota (FM1 and FM2) incubated in *in vitro* digesters under anaerobic conditions. Redox potential, SCFA, and gas and nitrite production were monitored (Fig. 1).

First, during the stabilization period for the four digesters, the redox potential plateaued around −245 mV (Fig. 1A). Each NO pulse then immediately increased the redox potential. At 10 μM NO, the pulses induced redox potential shifts of a very low magnitude. However, strikingly, the 100 μM NO pulses led to a 100-mV increase, and this oxidized state lasted for 3 to 5 h. It then progressively decreased to 15 mV higher than the initial baseline. The second and third NO pulses showed less impact on the redox potential, with a faster return to low values around −230 mV, which remained 15 mV higher than before the pulses.

Second, the SCFA production was measured after NO exposure (Fig. 1B). The 10 μM NO exposure induced a transient impact on SCFA production for FM2, characterized at 24 h by a slight increase of acetate proportion and a decrease of butyrate proportion. Following 100 μM NO exposure, the proportion of acetate remained stable, while the proportion of propionate increased over time and the butyrate proportion decreased.

Third, samples were also collected daily from the atmospheric phase of the bioreactor to evaluate gas composition and to ascertain anaerobic conditions (Fig. 1C). The excess of gas produced was also measured before and after NO exposure (Fig. 1D). This highlighted that FM1 was a CH_4_-excreting subject and that the methane proportion decreased following NO exposure. In contrast, CO_2_ increased with NO exposure and H_2_ was detected from 24 h onwards. FM2 gas composition remained rather similar at the lower NO dose, but for 100 μM NO, N_2_ proportion increased after 24 h, and H_2_ was detected from 24 h onwards (Fig. 1C). In addition, as an indicator of a lower fermentation activity, the gas volume exceeding the pressure within the digester vessels dropped after NO exposure in a dose-dependent manner: for FM1, gas volume decreased from 565 ± 83 ml to 414 ± 93 ml following 10 μM NO and reached 253 ± 74 ml following 100 μM NO. For FM2, gas volume decreased from 250 ± 36 ml to 139 ± 146 ml and to 108 ± 40 ml for 10 μM and 100 μM NO, respectively (Fig. 1D).

Fourth, nitrite concentration increased sharply upon each NO addition (Fig. 1E). Strikingly, for both FM1 and FM2 conditions, nitrite concentration increased following 100 μM NO in a pulse dependent-manner, reaching a maximum 1 h after NO addition and being barely detected 6 h after each NO pulse. The first 100 μM NO pulse led to the highest nitrite concentrations of 200 μM and 575 μM, respectively, for FM1 and FM2. The second 100 μM NO pulse led to nitrite concentration peaks of 475 μM and 175 μM and the third pulse to a maximum of 275 μM nitrite for FM2 and 100 μM for FM1. For both FM1 and FM2, nitrite concentrations were down to 0 μM 6 h after each NO pulse. For the 10 μM NO, the nitrite concentrations also followed the pulses but the peaks were less visible. Altogether, these results highlighted changes within fermentation activities of the gut microbial communities following repetitive NO pulses.

### NO impacts 16S rRNA gene microbial diversity in a dose- and pulse-dependent manner.

**FIG 2:**
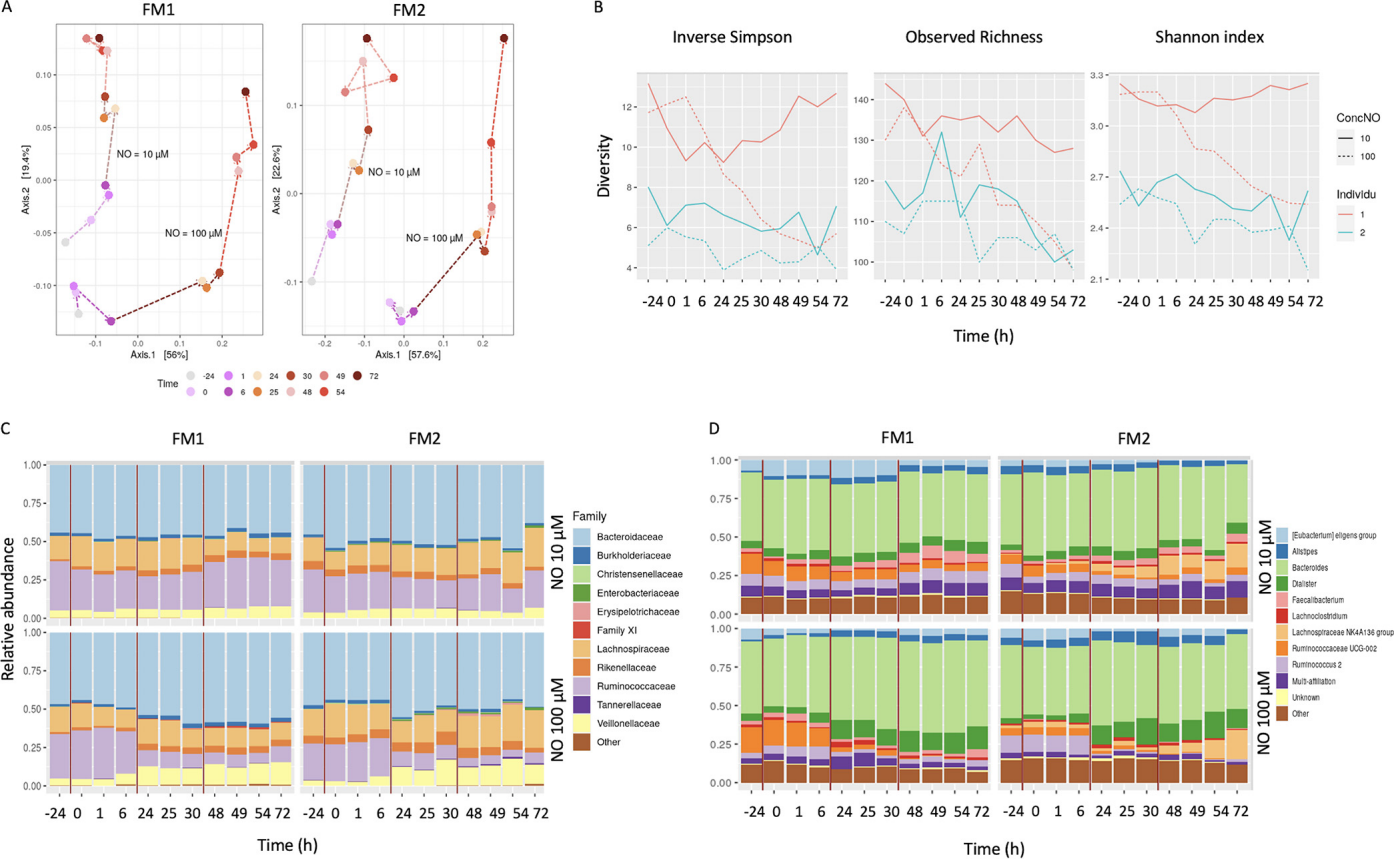
NO impact on the 16S rRNA gene microbial diversity. The impact of 10 μM and 100 μM NO administered in 3 pulses on two fecal microbiota (FM1 and FM2) was measured in terms of overall shift in microbial diversity by a principal-component analysis (A) and inverse Simson, observed richness, and Shannon index analysis (B). The relative abundance of the main families (C) and genera (D) was calculated.

The impact of NO on microbial diversity was analyzed by 16S rRNA gene sequencing (Fig. 2). NO modified the overall microbiome composition in a time-, pulse- and dose-dependent manner (Fig. 2A). The first NO pulse showed a drastic effect with a strong shift in PCA plot between 1 h and 24 h, a slightly less marked effect of the second pulse, from 25 h to 48 h, and a limited effect of the third pulse, from 49 to 72 h. The microbial diversity of both FM1 and FM2 also decreased in a time- and NO dose-dependent manner (Fig. 2B).

At the family level (Fig. 2C), the relative abundance of *Ruminococcaceae* decreased, especially after the second and third 100 μM NO pulses, while the relative abundance of *Bacteroidaceae*, *Veillonellaceae*, and *Enterobacteriaceae* slightly increased. At the genus level (Fig. 2D), the abundance of *Ruminococcus* UCG-002 and *Ruminococcus* 002 decreased after the second and third 100 μM NO pulses. In contrast, *Bacteroides* and *Dialister*, a member of the *Veillonellaceae* family, increased in a dose-dependent manner. Sulfate-reducing bacteria such as Desulfovibrio vulgaris, which converts NO to nitrates, were not detected in the 16S rDNA. Similarly, members of the *Nitrosomonas*, *Nitrobacter*, *Subdoligranulum*, *Akkermansia*, *Nitromonas*, *Nitrobacter*, Streptococcus, and Pseudomonas genera were not detected.

**FIG 3:**
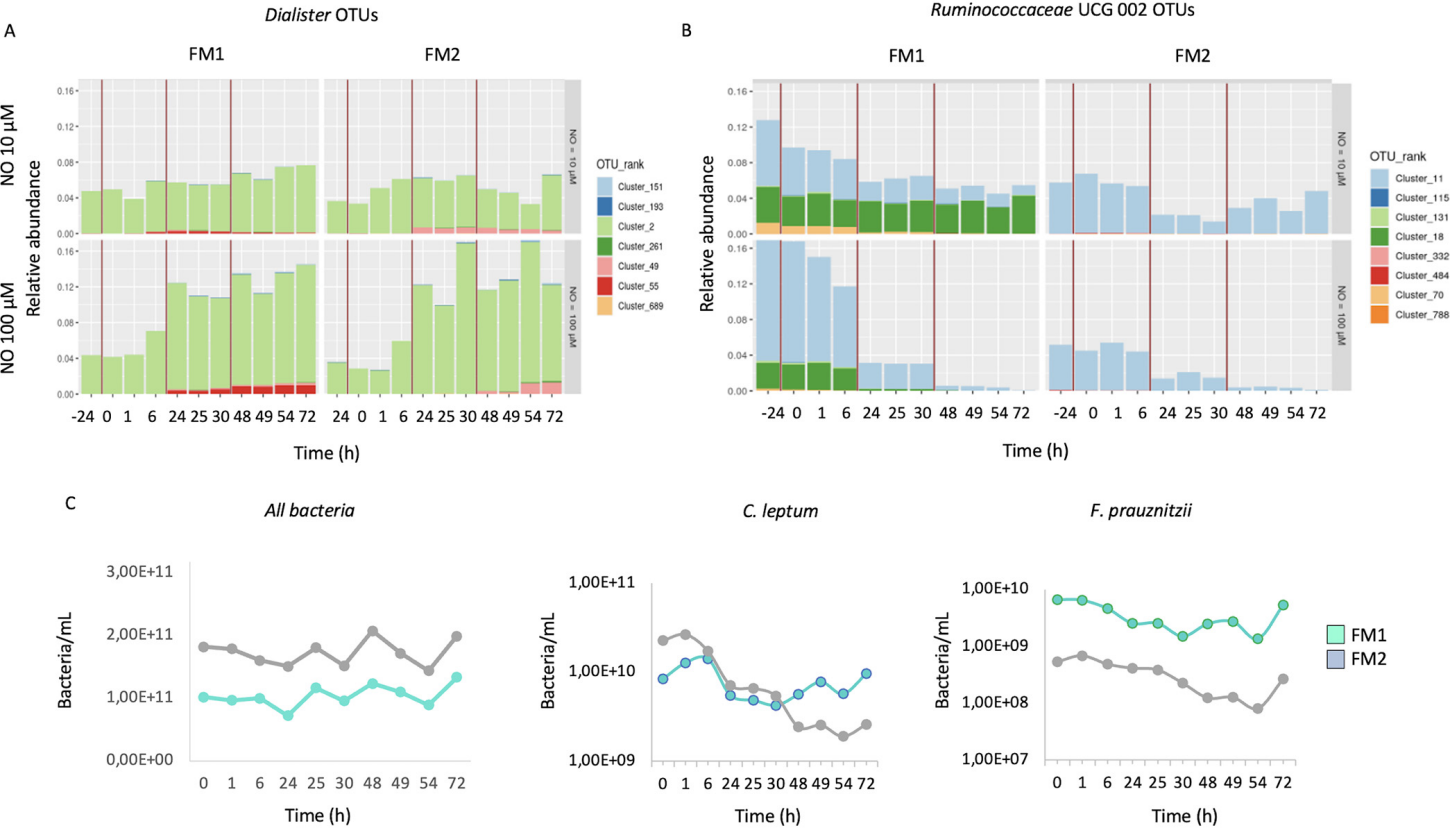
NO impact on the microbial diversity at the OTU level. The impact of 10 μM and 100 μM NO administered in 3 pulses on two fecal microbiota (FM1 and FM2) was measured on the shift in the relative abundance of *Dialister* (A) and *Ruminococcus* (B) OTUs. The impact of 100 μM NO pulses was also assessed for *C. leptum*, *F. prausnitzii*, and *Eubacteria* by qPCR (C).

The pulse effect was even more striking at the operational taxonomic unit (OTU) level, where most taxa changed between the first and second pulses rather than between the second and third pulses (not shown). Out of the 7 OTUs from the *Dialister* genus, one (cluster 2) showed a sharp abundance increase, and two (clusters 49 and 55) increased slightly (Fig. 3A). Within *Ruminococcaceae* UGC-002, cluster 11 decreased after each NO pulse, becoming almost undetectable after the third 100 μM NO pulse.

The impact of NO on specific groups was measured by quantitative PCR (qPCR). The bacterial biomass, as quantified by the *Eubacteria* probe, was not drastically impacted by NO exposure, with even 100 μM NO, leading to minor changes (8.1 ×  10^10^ to 5.7 × 10^10^ bacteria/ml) (Fig. 3C). In contrast, the *C. leptum* group, from the *Firmicutes* phylum, decreased from 2.3 × 10^10^/ml to 1.9 × 10^9^/ml after the 100 μM NO pulses (Fig. 3C). Likewise, Faecalibacterium prausnitzii decreased upon 100 μM NO pulses from 5.3 × 10^8^/ml to 8.0 × 10^7^/ml. No evident variations were identified for the subdominant groups *Lactobacillus/Pediococcus/Leuconostoc* and *Bifidobacteriaceae* (data not shown).

To complete those data, the microbiota of a third individual, FM3, was incubated with increasing concentrations of NO (0 to 500 μM) in a batch experiment, and microbiota diversity was analyzed by 16S rRNA gene sequencing (see Fig. S1 and S2 in the supplemental material). Consistent with data obtained in the fermenters, NO concentration and incubation times demonstrated an increased representation of the *Enterobacteriaceae* and *Lactobacillaceae* families and a decrease of *Ruminococcaceae*. The capacity of members of the *Lactobacillus* genus to sustain high NO concentrations was also confirmed. Thus, our *in vitro* batch experiments led to a strong effect of NO, providing an additional confirmation, under different experimental settings.

10.1128/mSystems.00558-21.1 FIG S1 Experimental settings detailing subjects, NO concentrations, and incubation conditions. Download FIG S1, JPG file, 0.6 MB. Copyright © 2021 Leclerc et al. 2021 Leclerc et al. https://creativecommons.org/licenses/by/4.0/ This content is distributed under the terms of the Creative Commons Attribution 4.0 International license.

10.1128/mSystems.00558-21.2 FIG S2 Selected data on the impact of NO on the microbiota composition of FM3, pointing out the congruence with continuous digesters’ results. Download FIG S2, JPG file, 0.7 MB. Copyright © 2021 Leclerc et al. 2021 Leclerc et al. https://creativecommons.org/licenses/by/4.0/ This content is distributed under the terms of the Creative Commons Attribution 4.0 International license.

### NO impacts the overall microbial metabolome.

**FIG 4:**
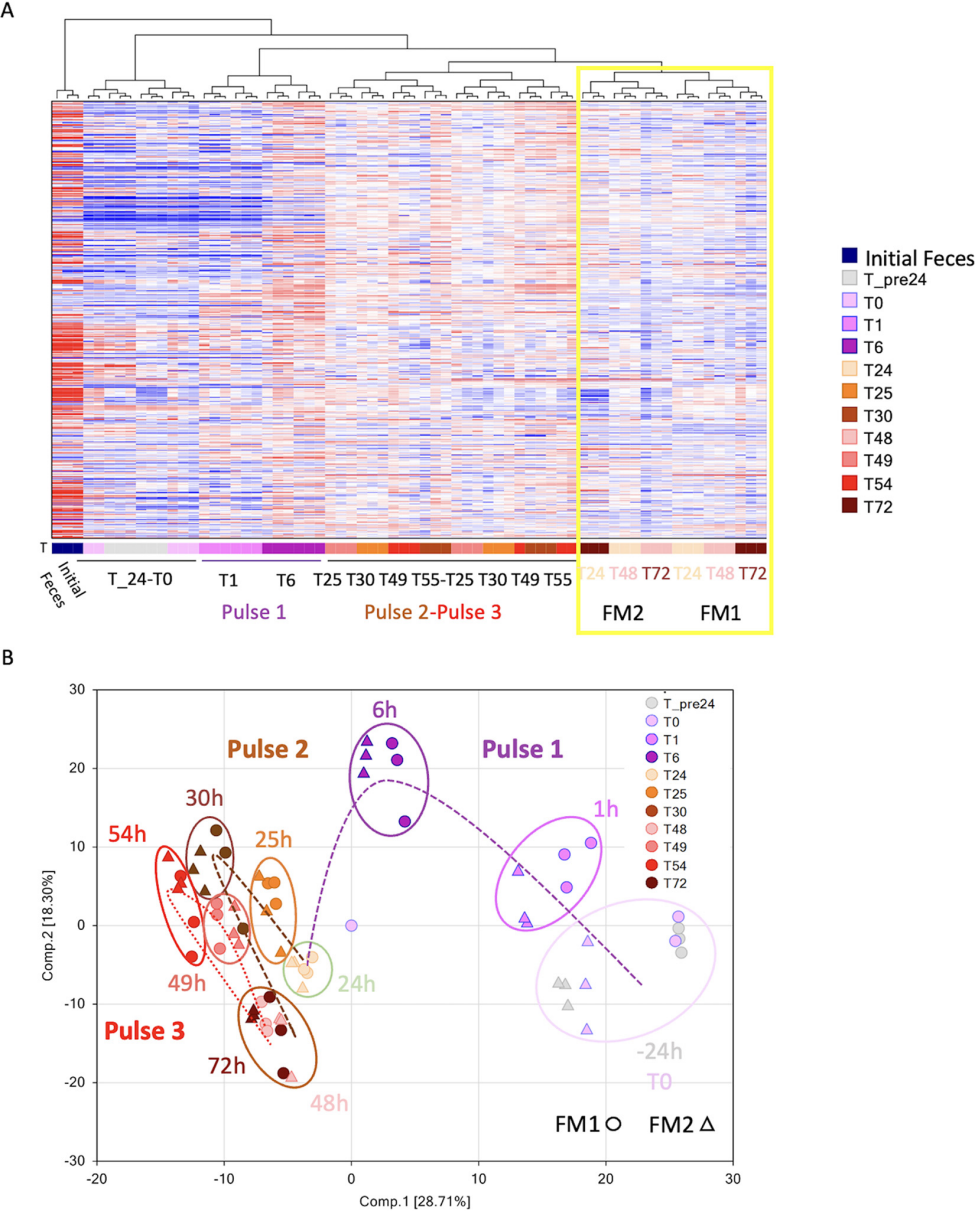
NO impact on the microbial metabolome. The impact of 100 μM NO administered in 3 pulses on two fecal microbiota (FM1 and FM2) was measured on the overall metabolome. Hierarchical clustering was used to provide an overview of the impact of NO in terms of time, main pathways, and individual differences. The yellow box highlights how FM1 and FM2 cluster together at the end of each pulse at 24, 48, and 72 h (A). The impact of NO pulses on the 529 metabolites found in FM1 and FM2 was analyzed by a principal-component analysis (B).

To further investigate the impact of NO on the ecosystem homeostasis, we performed untargeted metabolomics analysis following 100 μM NO pulses, as the major changes in microbiota diversity and composition were observed at this dose. For the two individuals, the overall metabolomics responses displayed strong responses to NO (Fig. 4). The hierarchical clustering of all metabolites provides a visualization of the time and pulse effects. The first NO pulse induced the strongest effect, and a shift in the overall microbiome is observed between 1 and 6 h (Fig. 4A). The second and third 100 μM NO pulses led to similar effects, and it is noteworthy that at the end of each pulse, at 24, 48, and 72 h, FM1 and FM2 metabolomes clustered together (Fig. 4A, yellow rectangle).

The microbial anaerobic metabolism reactions were also analyzed by plotting a principal component analysis (PCA) on the 529 metabolites identified (Fig. 4B). The PCA plot highlighted the broad metabolic impact of the 100 μM NO pulses. From the stabilization period to time 0 h (T0), each metabolome seemed quite stable. The first pulse at 1 h and, more strikingly, at 6 h commonly shifted the metabolome along the two components of the PCA. The second and third pulses shifted the metabolomes further away from the start but with less drastic changes than the first pulse. Interestingly, both metabolomes were clustered together after the second and third pulses, showing that NO has a strong and long-term impact on the metabolome. These overall changes in metabolomes followed the same trends observed for the microbiota diversity shift.

**FIG 5:**
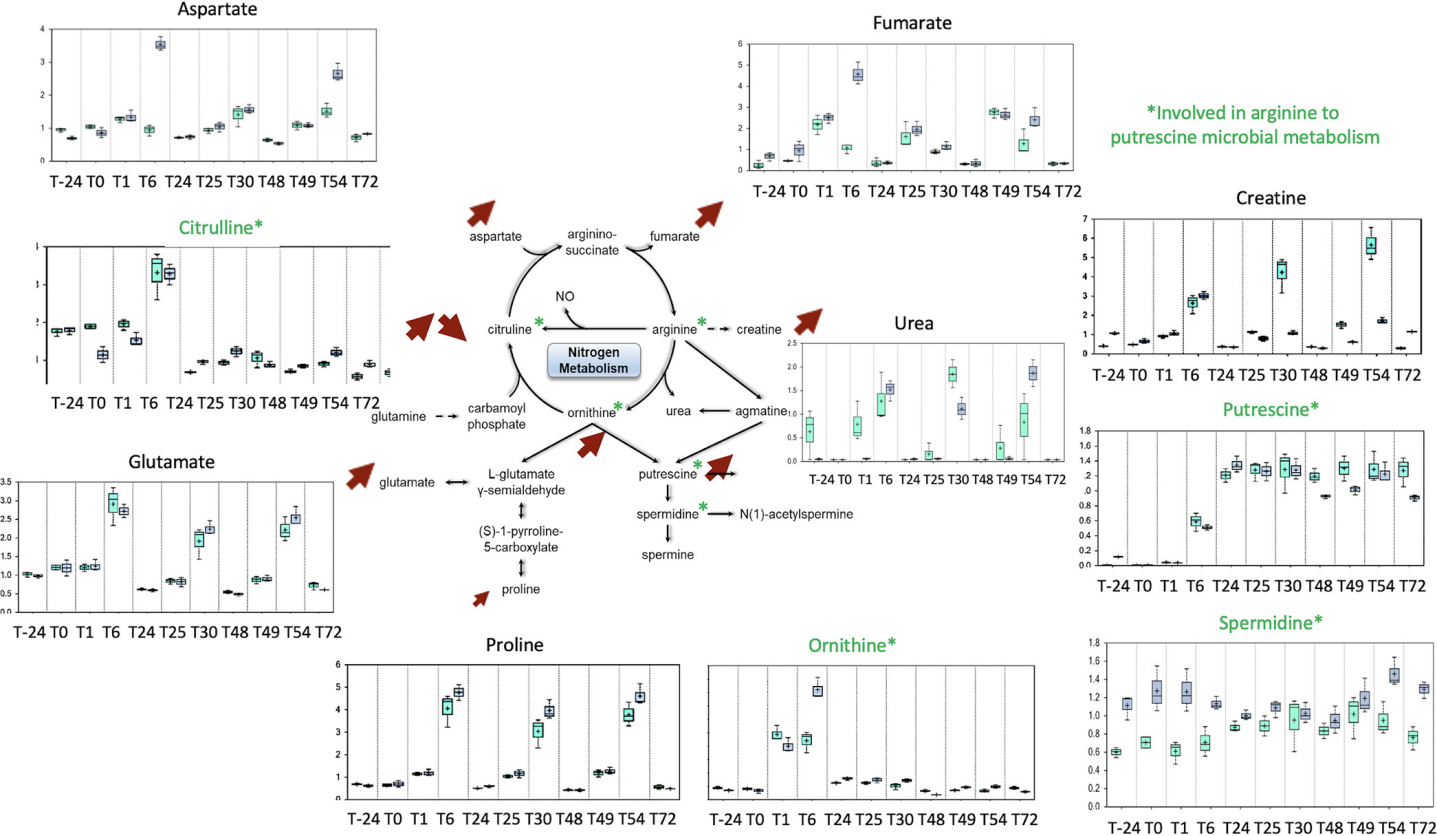
NO impact on the nitrogen cycle. The impact of 100 μM NO administered in 3 pulses on two fecal microbiota (FM1 and FM2) was measured on the main metabolism pathways involved in the nitrogen cycle. Metabolites known to be involved in gut microbial metabolism of arginine to putrescine are in green. Arrows indicate the up and down shifts of the corresponding metabolism. The *y* axis is scaled intensity, data normalized for biomass difference using Bradford assay. The box plots include the median (horizontal bar) and the mean (+). The top and bottom of the plots are the limits of the upper and lower quartiles. The dotted vertical lines correspond to the range (minimum and maximum) of the data distribution.

A detailed analysis highlighted a shift in the nitrogen cycle within the digesters (Fig. 5). Three profiles were observed. The first one, exemplified by citrulline and ornithine, showed a peak in production following the first NO pulse and a return to basal level afterwards. The second profile was characterized by a transient increased production following each pulse, like for aspartate, fumarate, creatine, glutamate, and proline. The last profile was a definitive increase of putrescine production from 6 h onwards. More precisely, the metabolites highlighted in green reflect the known metabolic pathway from asparagine to putrescine by the gut microbiota: pulses in citrulline and ornithine, followed by an accumulation of putrescine. In FM1 and FM2, spermidine concentration remained high compared to what is expected under anaerobic conditions.

**FIG 6:**
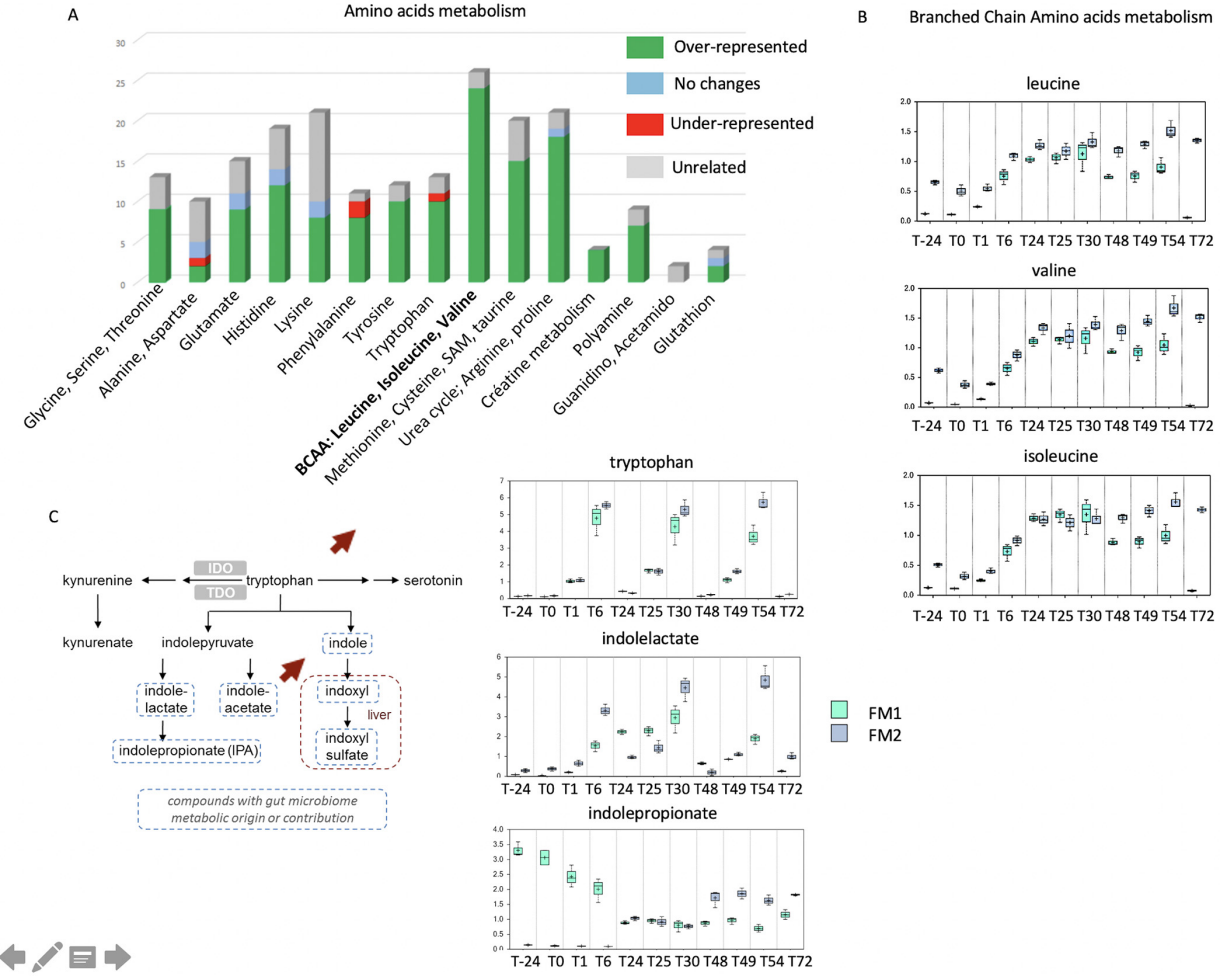
NO impact on the amino acid metabolism. (A) The impact of 100 μM NO administered in 3 pulses on two fecal microbiota (FM1 and FM2) was measured on the main amino acid metabolism, and changes were analyzed as under- or over-represented compared to initial stage. Branched-chain amino acid (B) and tryptophan (C) metabolism changes were specifically analyzed. Arrows indicate the up and down shifts of the corresponding metabolism. The *y* axis is scaled intensity, data normalized for biomass difference using Bradford assay. The box plots include the median (horizontal bar) and the mean (+). The top and bottom of the plots are the limits of the upper and lower quartiles. The dotted vertical lines correspond to the range (minimum and maximum) of the data distribution.

Among the amino acids, the most stimulated pathways were the BCAA (branched-chain amino acids) leucine, valine, and isoleucine, sharing common biosynthetic pathways and all stemming from intermediates of pyruvate metabolism (Fig. 6A and B). The tryptophan metabolism, shown with its links *in vivo* to serotonin documented as the gut-brain axis, increased in a pulse-dependent manner (Fig. 6C). Furthermore, its derivatives were also modified, since the concentration of indole lactate increased after each pulse while the production of the anti-inflammatory indole propionate decreased.

**FIG 7:**
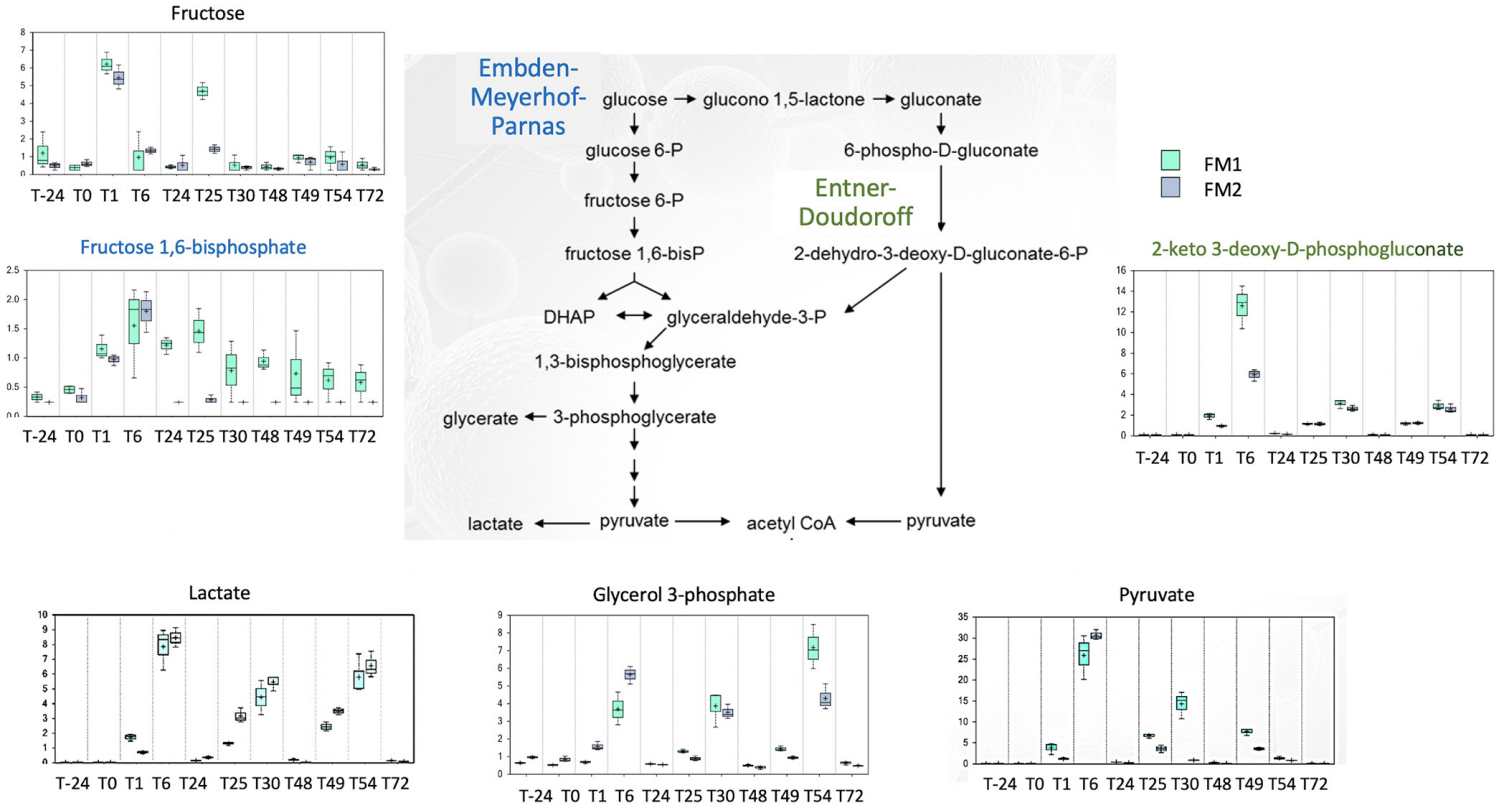
NO impact on pyruvate metabolism. The impact of 100 μM NO administered in 3 pulses on two fecal microbiota (FM1 and FM2) was measured on pyruvate metabolism. Metabolites specific to the Entner-Doudoroff and Embden-Meyerhof-Parnas pathways are colored in green and blue, respectively. The *y* axis is scaled intensity, data normalized for biomass difference using Bradford assay. The box plots include the median (horizontal bar) and the mean (+). The top and bottom of the plots are the limits of the upper and lower quartiles. The dotted vertical lines correspond to the range (minimum and maximum) of the data distribution.

The two main fermentation routes from pyruvate, the Embden-Meyerhof-Parnas pathway and the Entner-Doudoroff pathway, were impacted upon NO exposure (Fig. 7). Pyruvate concentration sharply increased after the first pulse, while the second and third NO pulses showed less effect. Concomitantly, the concentrations of several sugar monomers increased: fructose increased after the first pulse, while fucose increased after each of the three NO pulses. The accumulation of glycerol-3-phosphate, common to both pathways, was pulse dependent. Interestingly though, fructose 1,6 bisphosphate and 2-keto 3-deoxy-d-phosphogluconate, specific for EMP and ED pathways, respectively, increased after NO. Lactate concentration also increased transiently following each NO pulse, while the short-chain and medium fatty acids decreased. NO led first to an accumulation of pyruvate, with less SCFA but more lactate being produced.

### Escherichia coli, Proteus mirabilis, and Enterococcus faecalis strains isolated after NO pulses are resistant to NO.

**FIG 8:**
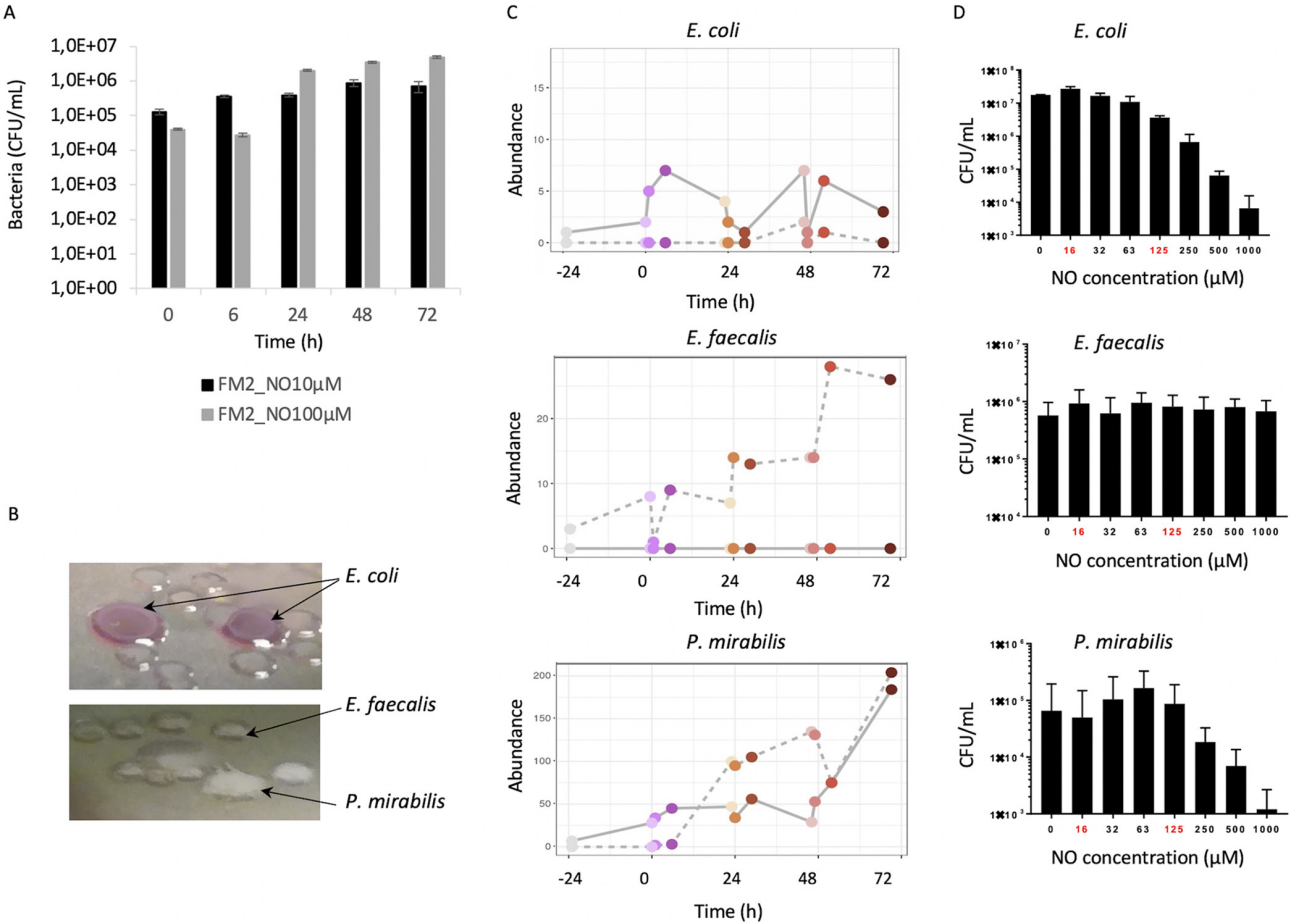
NO impact on E. coli, P. mirabilis, and E. faecalis strains isolated after NO pulses. Gram-negative enteric bacterial populations were isolated following 10 μM and 100 μM NO administered in 3 pulses on FM2 microbiota, enumerated (A), and identified (B) on MacConkey selective medium. Their corresponding abundance was measured by 16S rDNA sequencing (C). The isolated strains were then subjected to *in vitro* NO stress at various concentrations (0 to 1,000 μM), and survival was assessed by plating on selective media (D).

To correlate this potential metabolic shift from anaerobic to facultative anaerobic with the increased abundance of aerotolerant bacteria measured by 16S rRNA gene sequencing, we enumerated the Gram-negative enteric bacteria remaining after NO pulses by plating on selective media. The bacterial concentration increased from 4.0 × 10^4^ CFU/ml before the 100 μM NO stress up to 4.9 × 10^6^ CFU/ml after the third pulse (Fig. 8A). The enumeration results at 10 μM NO were less marked, with an increase from 1.3 × 10^5^ CFU/ml up to 9 × 10^5^ CFU/ml.

Numeration on specific media revealed distinct colonies morphotypes, which were further identified by Vitek MS as strains belonging to Escherichia coli, Enterococcus faecalis, and Proteus mirabilis (Fig. 8B).

To go further, we analyzed whether the OTUs corresponding to those strains exhibited any abundance differences over time (Fig. 8C). E. coli*-Shigella* OTU160 displayed a pulse effect and increased over time. E. faecalis OTU 158 abundance showed significant increases following each pulse and reached 30 times its initial relative abundance. For P. mirabilis OTU 41, a pulse and dose response were measured, with up to 200 times its initial abundance after the third pulse.

The isolated E. coli, E. faecalis, and P. mirabilis strains were then grown *in vitro*, and survival after NO exposure (0 to 1,000 μM) was measured by plating (Fig. 8D). E. coli exposure to NO up to 63 μM did not significantly change survival, but survival decreased by 1 log at higher concentrations. E. faecalis survival was not affected by NO concentrations up to 1,000 μM. P. mirabilis could sustain NO exposure up to 125 μM before a significant drop in survival at 250 μM and above.

## DISCUSSION

Using *in vitro* fermentation models to mimic the human colonic gut environment, our data provided key elements regarding the effects of repetitive NO pulses on the microbiome. We showed that NO induced both transient and long-term modifications of the gut environment, microbial composition, and metabolism.

Very limited data are available on *in vivo* NO concentrations. To support our choice of 10 and 100 μM, a study based on chemiluminescence technique in patients suffering from ulcerative colitis revealed that NO concentrations would be more than 100 times higher in inflamed colonic luminal content than in healthy individuals (8). Although this study does not consider the source of NO production (epithelial cells, macrophages, and bacteria), a predictive mathematical model simulating NO diffusion and reaction in colonic crypts determined that NO concentration reached about 0.2 μM (9). The 10 μM and 100 μM doses might not mimic a *bona fide* intestinal inflammation but might accentuate the influencing effects on the human gut microbiota. The higher doses used for the *in vitro* batch and the isolated strain cultures highlighted how individual species and strains can sustain and grow under high NO concentration over a long period of time, as opposed to facing short pulses.

### NO impacts the ecosystem redox potential.

The redox potential in the *in vitro* fermentation system was stabilized before NO stress at approximately −245 mV, close to the physiological oxidation status of the intestinal lumen (16). Each NO pulse immediately shifted the overall redox potential, with the second and third pulses leading to short-lasting redox potential increases, suggesting a resilience of the intestinal ecosystem. The overall redox potential indicates that an addition of NO immediately changed the redox state of one or more redox couples within the microbial communities. Actually, hydrogen disposal reactions were inhibited by the higher redox potential, as evidenced by the decrease in methane production and the increase in hydrogen in the fermenter gas phase. These changes could be correlated with transient disruption of bacterial syntrophy between H_2_ and CO_2_ producers and obligatory hydrogenotrophic *Archaea* methanogens (17).

Hydrolysis and fermentation processes rely on a balance of redox reactions allowing the reoxidation of mandatory cofactors such as NADP (18). A disruption of redox couples can dramatically affect enzymatic activities, since redox couples function as ON/OFF switch regulation of redox sensitive proteins containing cysteine or methionine catalytic sites (16). To go further, such an oxidation of the gut environment drives a shift of other fermentation metabolites, which constitute alternative terminal electron acceptors. Changes in final electron acceptors, such as nitrate ions, sulfate ions, or even carbon dioxide, may support the growth of bacteria using anaerobic respiration as an energy supply (15, 19). Accordingly, addition of NO may represent an exogenous source providing terminal electron acceptors through reactive nitrogen species reduction for anaerobic respiration. Interestingly, at 1 h after each 100 μM NO pulse, nitrite concentrations increased prior to reaching zero concentrations. N_2_ does not drastically accumulate in the gas phase but only slightly increased after the first pulse, suggesting that denitrification did not happen in the digester. Hence, the oxidation level, upon the pulses, increased from NO to nitrite in the nitrogen cycle. The NO and nitrite pulses could result from the fast oxidation and consumption of NO efficiently used as an exogenous energy/oxidation source. These results suggest an adaptation of microbial populations to this exogenous source that represents NO.

### NO changes the microbial ecosystem structure and metabolism.

NO modified the overall anaerobic fermentation pathways. The human gut is an environment where N_2_ accounts for 60 to 80% of the gaseous content (20). In the digesters, the volumes of gas resulting from fermentation activities dropped in a NO dose-dependent manner, including N_2_ and CO_2_ gases. Although the digesters provided stable conditions to assess NO pulses without any confounding factors, such as dietary nitrogen intake, the nitrite concentrations in the digester were much higher than that of the equivalent introduced NO. NO is produced from the amino acid arginine by many bacterial species via the enzyme nitric oxide synthase (NOS) (21). Furthermore, the higher redox potential, deleterious for strict anaerobes, is consistent with the accumulation of putrescine in the digesters following pulses of citrulline and ornithine. Consistent with this, NO modified amino acid composition and concentration in the fecal microbiota. The branched-chain essential amino acids valine, isoleucine, and leucine, which sharply increased upon NO pulses, have been linked to metabolic disorders such as insulin resistance and type 2 diabetes (22). In addition, deamination of amino acids leads to lactate or succinate, and both were increased after each high NO dose. Conversely, NO led to a decreased concentration of SCFA from acetate and butyrate and also longer fatty acids, such as caproic, caprylic, and capric acid (C10:0). In addition, because of the lack of strict anaerobes, the disruption of both the Embden-Meyerhof-Parnas and the Entner-Doudoroff pathways, and the hydrogen-consuming reactions, fermentation patterns are expected to be shifted. However, ^13^C and ^15^N isotope experiments would be necessary to confirm these metabolic shifts.

### NO shifted the microbiota composition.

*Dialister* genus, from the *Veillonellaceae*, was favored by NO. Members of the *Dialister* genus are asaccharolytic but produce lactate and decarboxylate succinate to propionate (23). Consistent with this, increased propionate production was measured upon NO pulses. Propionate production through the succinate pathway has been described for members of the *Bacteroides* and *Dialister* genera. As a matter of fact, both malate and fumarate concentrations increased upon NO pulses (Fig. S3). Hence, we can suggest a link between the abundance of *Dialister* OTUs and the increased propionate production. *Dialister* isolates originate from gut human clinical samples, and this genus has been suggested as a potential biomarker of activity in IBD-associated spondyloarthritis (24). Concomitantly, a depletion of butyrate production, as observed in our study, could accentuate the pathological state, since this microbial-derived metabolite was associated with anti-inflammatory properties (25).

10.1128/mSystems.00558-21.3 FIG S3 Metabolites from the propionate pathway. Download FIG S3, TIF file, 2.6 MB. Copyright © 2021 Leclerc et al. 2021 Leclerc et al. https://creativecommons.org/licenses/by/4.0/ This content is distributed under the terms of the Creative Commons Attribution 4.0 International license.

### NO stress increased pathobionts from the *Enterobacteriaceae* and *Gammaproteobacteria*.

NO favored the emergence of putative pathobionts, and we isolated strains from E. coli, P. mirabilis, and E. faecalis species. We further showed that these strains sustained growth under high NO concentrations. The higher doses we used for the *in vitro* batch and the isolated strains highlighted how individual species and strains can sustain and grow under high NO concentrations over a long period of time as opposed to facing short pulses as in the continuous digesters. Batch conditions also increased the relative abundance of *Proteobacteria* and the representation of the genera Escherichia and *Shigella*, being overrepresented at 500 μM NO. Although batch cultures do not represent gut-associated microbial community dynamics, these data further suggest that NO stress favors the selection of pathobionts in the gut microbiota. Several pieces of evidence already exist in the literature regarding pathobionts in inflammatory bowel diseases. A microbial signature pattern associated with inflammation is recurrently described by the depletion of many oxygen-intolerant prokaryotes, while a small number of aerotolerant and potentially pathogenic prokaryotes emerge, such as E. coli and E. faecalis (15, 26, 27). NO could stimulate the growth of microorganisms presenting the metabolic capacity to use microbial-derived nitrosative products as substrates. Competition from N-derived substrates between commensals and pathogens, under higher redox potential, is often described in the context of inflammation (15). In addition, propionate production through the propane 1-2 diol pathway, described for pathogens such as E. coli, Salmonella, Ruminococcus torques, and E. faecalis, involved fucose through fuculose, l-lactaldehyde, and propane 1-2 diol. Consistently, we detected a peak of fucose production after each NO pulse. The increased prevalence and isolation of NO-resistant E. coli and E. faecalis could also account for propionate production through this second pathway (28). Moreover, the increase of these pathobiont strains might be amplified by the depletion of butyrate produced by *Firmicutes*, which is associated with protective roles against *Proteobacteria* through growth inhibition (25–27). We have consistently observed a 1-log decrease in F. prausnitzii following NO stress. Furthermore, Eubacterium eligens, another butyrate-producing member of the *Clostridium* cluster XIV (*C. leptum* group) (29), was also decreased upon NO stress. A shift in F. prausnitzii abundance has been associated with unbalanced microbiota and increased gut inflammatory diseases (30).

Taken together, we have shown that NO drastically impacts gut microbiota diversity and function and might favor the selection of pathobionts. The oxidation of the gut environment drives the microbial communities toward a depletion of strict anaerobes, and alternative uses of amino acids and nitrogen cycles appear. The depletion of immunomodulating species, such as *F. prausnitzii*, *R. intestinalis*, and *E. eligens*, probably participate in aggravating the inflammatory response. Indeed, OTUs that appeared less abundant upon NO pulses, such as *F. prausnitzii*, are known for their low abundance in IBD patients’ microbiota. In addition, previous studies focusing on redox potential shifts, fermentation activities, and microbial composition associated with IBD show similarities with our study (15, 30). This suggests that NO influences the ecosystem stability, leading to a “dysbiosis” state that presents similarities with the microbiota of IBD patients. NO may be a pathophysiological factor leading to microbial signature of gut dysbiosis, among other multifactorial factors associated with the host.

### Conclusions.

The disruption of gut microbiota homeostasis has been associated with numerous diseases, and a disproportionate inflammatory response, including overproduction of nitric oxide (NO) in the intestinal lumen. However, the influence of NO on the human gut microbiota has not yet been fully characterized.

In this study, we highlight that NO quickly changed microbial fermentations, taxonomic composition, and metabolomes of the gut microbiome. We furthermore show that NO induces both transient and long-term modifications of the gut environment and microbial ecosystem. Finally, newly isolated E. faecalis, P. mirabilis, and E. coli strains sustain growth under high NO concentrations. NO over time shifts the trophic chain to conditions that are unfavorable for strict anaerobic microbial processes, implying that a prolonged or uncontrolled inflammation has detrimental and irreversible consequences on the human microbiome. The depletion of immunomodulating species such as *F. prausnitzii* and the concomitant increase of pathobionts such as E. faecalis suggest that NO influences the ecosystem stability, leading to a dysbiosis state that presents similarities with microbiota of IBD patients.

We believe that our findings on the impact of NO on the microbiome ecosystem improve our understanding of the intricate relationship between the host immune response and its microbiota.

## MATERIALS AND METHODS

### Feces collection.

Three fresh fecal samples from unrelated healthy adults were collected and immediately placed at −80°C without any additives. Subject 1 was a methane-producer female, subject 2 was a male non-methane-producer, and subject 3 was a non-methane-producer female.

Fresh fecal materials from subject 1 and 2 were used to inoculate ARCOL (artificial colon) from the unrelated healthy adults, who had no history of gastrointestinal disease and no antibiotic or no probiotic therapy for the last 3 months prior to the study. This study is a noninterventional study with no additions to usual clinical care. The protocol does not require approval from an ethics committee according to the French Public Health Law (CSP Art L 1121-1.1).

Immediately after defecation, fecal samples were collected into sterile plastic containers and placed in an airtight anaerobic box (GENbag anaerobic gas pack systems; bioMérieux, France), transported, and processed at the laboratory within 6 h. Fecal microbiota suspensions, named FM1 and FM2 for subjects 1 and 2, respectively, were sequentially prepared under strict anaerobic conditions after transfer in an anaerobic Freter-type chamber (COY Laboratory Products Inc., USA) with an atmosphere containing a gas mixture of 90% N_2_, 5% CO_2_, and 5% H_2_ (Air Products, France). About 30 g of stools was resuspended in 100 ml of sterile sodium phosphate buffer (30 mM, pH 6.5) and filtered through a 500-μm-diameter Inox sieve (Retsch, Germany).

Subject 3 fresh fecal sample was collected and prepared under strict anaerobic conditions after transfer in an anaerobic Freter-type chamber (COY Laboratory Products Inc., USA) with an atmosphere containing a gas mixture of 90% N_2_, 5% CO_2_, and 5% H_2_ (Air Products, France). About 30 g of stools was resuspended in 100 ml of anaerobic sterile sodium phosphate buffer (30 mM, pH 6.5). The fecal microbiota named FM3 was used to inoculate Balch tubes (1/10) (Bellco) in BHI.4 medium and exposed to NO at final concentrations from 0 μM to 1,000 μM for 48 h at 37°C. The protocols for the continuous digesters and *in vitro* batch incubations of FM3 and newly isolated strains are summarized in the supplemental material (Fig. S1).

### *In vitro* fermentation system.

The ARCOL continuous fermentation system (MiniBio Reactors; Applikon, Netherlands) is a dynamic and computer-controlled model simulating the human luminal colonic environment (31). The system consists of a pH- and temperature-controlled main bioreactor reproducing the luminal colonic medium. The nutritive colonic medium was continuously introduced into the main bioreactor to simulate sequential input of ileal effluents as described in reference 32, with slight modifications. Anaerobiosis inside the bioreactor was maintained by the sole activity of resident microbiota. Redox potential was constantly measured using a redox sensor (Applikon, Netherlands) and corrected into an Eh value by adding 200 mV to the recorded values according to the manufacturer’s instructions. The ARCOL was set up to reproduce the mean conditions found in a healthy human adult colon with a total volume of 300 ml, a fixed temperature of 37°C, a constant pH of 6.2, a stirring speed at 400 rpm, and a mean retention time of 24 h.

The fermentation systems were inoculated with 100 ml of FM1 and FM2 first under batch conditions for 24 h and then allowed to stabilize under continuous conditions for 7 days. An additional 24 h was considered the final stabilization period prior to NO treatment.

### NO treatment and sampling.

Diethylamine NONOate (DEA NONOate) was chosen as the NO donor (Calbiochem Millipore Merck, USA). DEA NONOate decomposes spontaneously in solution to generate up to 2 molar equivalents of NO. NO solutions were resuspended into sterile deionized water just before use. Following the stabilization period, the NO donor was added to the fermentation systems at T0 at two final concentrations (10 μM and 100 μM) every 24 h during a period of 3 days. These 3 pulses (0, 24, and 48 h) allowed to compensate the continuous biomass dilution occurring within the bioreactor due to regular input of nutritive medium and output of fermentation medium. For each fecal microbiota FM1 and FM2, two sets of fermentation were performed, one inoculated with 10 μM NO pulses and one with 100 μM NO pulses. Each fermenter was set up to be its own control, and colonic medium samples collected on day 7 of the stabilization phase (T24) and just before NO exposure (T0) were considered control conditions.

Three hundred-microliter samples were collected in triplicates from the fermentation system through the medium outlet just before each NO pulse (T0, T24, and T48), as well as 1 h, 6 h, and 24 h afterwards (pulse 1, T1, T6, and T24; pulse 2, T25 and T30; T48; pulse 3, T49, T54, and T72). Samples were used for microbiota characterization (16S rRNA), metabolites (untargeted metabolomics and SCFA analysis), and species identification.

### Nitrite measurement.

Griess tests were performed to estimate accumulated total nitrite and nitrate contents within the colonic medium throughout the NO exposure. Fermenters’ contents were centrifuged and supernatants were filtered. Nitrite/nitrate concentrations were determined in the supernatants using the colorimetrical Griess reagent system (Promega, USA) as described by the manufacturer’s instructions. Blank samples were composed of the Griess reagent and supernatants of fresh nutritive medium.

The absorbance (optical density) was measured with a microplate spectrophotometer (Epoch; Biotek Instruments, USA) at 540 nm (Gen 5 2.00 software).

### Gas and SCFA analysis.

Gas overproduction in the atmospheric phase of ARCOL was collected daily into an external bag connected to the condenser outlet. Analysis of O_2_, N_2_, CO_2_, CH_4_, and H_2_ was performed using an HP 6890 gas chromatograph (Agilent Technologies, Santa Clara, USA) coupled with a micro-TCD detector (Agilent Technologies, Santa Clara, USA) as previously described (31).

The three major SCFAs (acetate, propionate, and butyrate) were quantified in colonic samples by high-performance liquid chromatography (HPLC) (Elite LaChrom; Merck Hitachi, USA) coupled with a DAD diode as described in reference 31.

### DNA extraction.

Two hundred microliters of collected colonic medium was thawed on ice, and total genomic DNA was extracted using the QIAamp PowerFaecal DNA kit (Qiagen, Germany) according to the manufacturer’s instructions. Quality and amount of the extracted DNA were evaluated using NanoDrop 2000 spectrophotometry (Thermo Scientific).

### qPCR.

The composition of the microbiota was analyzed by measuring *Eubacteria* and eight dominant and subdominant bacterial groups, taxa, and species (*Bacteroides/Prevotella*, Clostridium coccoides, Clostridium leptum, *Bifidobacteria*, *Lactobacillus/Leuconostoc/Pediococcus*, *Enterobacteriaceae*, Escherichia coli, and Faecalibacterium prausnitzii). Specific regions of the 16S rRNA genes were detected using specific primers and probes by real-time qPCR (Quantstudio 3; Applied Biosystem by Thermo Fisher Scientific, USA) with either TaqMan universal PCR 2× master mix (Applied Biosystems, USA) or with Takyon Rox SYBR MasterMix dTTP blue (Eurogentec, Belgium). The primers and methods are described in reference 33. Each reaction was run in duplicate, and qPCR experiments were performed according to the following method: 1 cycle at 95°C for 10 min and 40 cycles of 95°C for 15 s and 60°C for 1 min. For SYBR green amplification, a melting curve was added to show the amplification specificity. Standard curves of reference strains from bacterial genomic DNA were generated by plotting cycle threshold (*C_T_*) versus cell number of reference strain in the qPCR assay, as described in reference 33. Results are expressed as number of bacterial cells/milliliter of colonic medium (34).

### V3-V4 16S rRNA gene sequencing.

To explore the diversity and composition of the colonic microbiota following the repetitive NO donor pulses, the V3-V4 region of the 16S rRNA gene was amplified in the collected samples using the following primers (Eurofins Genomics, Germany): V3Forward (5′-CTTTCCCTACACGACGCTCTTCCGATCTACGGRAGGCAGCAG-3′) and V4Reverse (5′-GGAGTTCAGACGTGTGCTCTTCCGATCTTTACCAGGGTA-TCTAATCCT-3′). Each reaction was run in a final volume of 50 μl with a final concentration of 20 μM each primer and 20 ng of colonic DNA samples. PCR experiments were performed with 1 cycle at 94°C for 1 min and 30 cycles of 94°C for 1 min, 65°C for 1 min, and 72°C for 1 min. The expected size fragments were about 500 bp based on E. coli base pair numbering. The subsequent 46 V3-V4 DNA r16S amplicons were sent for MiSeq Illumina sequencing (MiSeq kit V2 2 × 250 bp) to the Genotoul sequencing platform (Castanet-Tolosan, France). Data quality checks and analysis were performed using the INRA-Migale server (35). Sequencing resulted in an average of 43,119 high-quality reads per samples (36,075 to 50,366).

### Analyses of microbiota composition and bacterial diversity.

Paired-end reads obtained from MiSeq sequencing were analyzed using the Galaxy-supported FROGS (find, rapidly, OTUs with galaxy solution) pipeline (36). Briefly, overlapping reads were merged using PEAR (37), and adapters were removed with cutadapt. Reads were then clustered with SWARM (38), chimera were detected with VSEARCH (39), and singletons and chimera were removed. SWARM does not use a fixed similarity threshold to pick OTUs but relies instead on an adaptive OTU definition strategy where (groups of) reads are iteratively grown by adding all reads at a distance at most 1 bp from the group, until no group can be grown anymore. All those steps followed the FROGS guidelines and resulted in a 82.4% read retention rate. Taxonomic assignment was performed against SILVA 132 (40), and OTUs with affiliations (<95% coverage or <95% identity) were filtered out. A total of 242 OTUs were obtained.

Diversity analyses were performed using phyloseq R package (41) in addition to custom scripts. Samples were rarefied to even sampling depths (11,898 reads/sample) before computing diversity metrics. We computed both within-sample alpha diversities (observed richness and Shannon) and between-samples compositional diversities (Bray-Curtis). Principal coordinate analysis (PCoA) was also performed on the Bray-Curtis distance matrix to obtain a two-dimensional representation of the samples. Data are at the SRA repository under Project number PRJNA706948.

### Metabolomics.

From the fermenters incubated with 100 μM NO, 23 samples were collected in triplicates throughout the experiment (before inoculation with fecal samples and during fermentation) and were immediately stored at −80°C. The samples were then sent on dry ice to Metabolon (Research Triangle Park, NC, USA). Samples were prepared using the automated MicroLab STAR system (Hamilton Company). To remove protein, dissociate small molecules bound to protein or trapped in the precipitated protein matrix, and recover chemically diverse metabolites, proteins were precipitated with methanol under vigorous shaking for 2 min (Glen Mills GenoGrinder 2000) followed by centrifugation. Samples were placed briefly on a TurboVap (Zymark) to remove the organic solvent. The sample extracts were stored overnight under nitrogen before preparation for analysis. The resulting extract was divided into 4 fractions that were analyzed as two separate reverse-phase ultrahigh-performance liquid chromatography-tandem mass spectrometry (RP/UPLC-MS/MS) methods with positive-ion mode electrospray ionization (ESI), one by RP/UPLC-MS/MS with negative-ion mode ESI and one by hydrophilic interaction chromatography/UPLC-MS/MS (negative ion mode ESI).

Analysis using the Metabolon DiscoveryHD4 platform for data processing and in-house database compound matching led to a total of 529 metabolites. Metabolomics data are in Table S1 in the supplemental material.

10.1128/mSystems.00558-21.4 TABLE S1 Metabolomics data. Download Table S1, XLSX file, 2.0 MB. Copyright © 2021 Leclerc et al. 2021 Leclerc et al. https://creativecommons.org/licenses/by/4.0/ This content is distributed under the terms of the Creative Commons Attribution 4.0 International license.

### Bacteria enumeration, isolation, and mass spectrometry-based identification.

Facultative anaerobic bacteria were isolated from FM2 following 100 μM NO pulses. Samples collected at 0 h, 6 h, 24 h, 48 h, and 72 h were diluted in sterile 0.9% NaCl solution, plated onto MacConkey (for the selection of *Enterobacteriaceae*) and d-Cocossel (for the selection of *enterococci* and group D streptococci) agar, and incubated at 37°C for 24 h under aerobic conditions. Bacterial CFU were counted and further streaked on Trypticase soy agar plates. Colony identification of the isolated strains was performed via Vitek MS (bioMérieux, France).

### *In vitro* NO stress on isolated bacterial strains.

Isolated strains identified as E. coli, P. mirabilis, and E. faecalis were grown to late exponential growth phase in LB medium at 37°C. Cultures were harvested and diluted 1:1,000 in RPMI medium (Gibco, France), and 150 μl was dispatched into 96-well plates. Bacteria were exposed to NO at final concentrations from 0 μM to 1,000 μM for 4 h at 37°C. Bacterial survival rate was quantified by plating on LB agar, and results are expressed as number of CFU/ml from three independent experiments done in duplicate. See the supplemental material (Fig. S1) for the incubation times and doses for the *in vitro* and continuous digesters conditions.

### Data availability.

16S rRNA gene data sets generated and/or analyzed during the current study are available in the SRA repository (www.ncbi.nlm.nih.gov/sra/docs/submitportal/) under BioProject number PRJNA706948. Metabolomics data are in Table S1.

10.1128/mSystems.00558-21.5 TABLE S2 16S rDNA main genera and OTUs. Download Table S2, XLSX file, 0.06 MB. Copyright © 2021 Leclerc et al. 2021 Leclerc et al. https://creativecommons.org/licenses/by/4.0/ This content is distributed under the terms of the Creative Commons Attribution 4.0 International license.
