# Supplementary figures and images for: Nitric Oxide Impacts Human Gut Microbiota Diversity and Functionalities
Source: mSystems. 2021 Sep 14;6(5):e00558-21. doi: 10.1128/mSystems.00558-21 (PMC8547463; doi:10.1128/mSystems.00558-21)

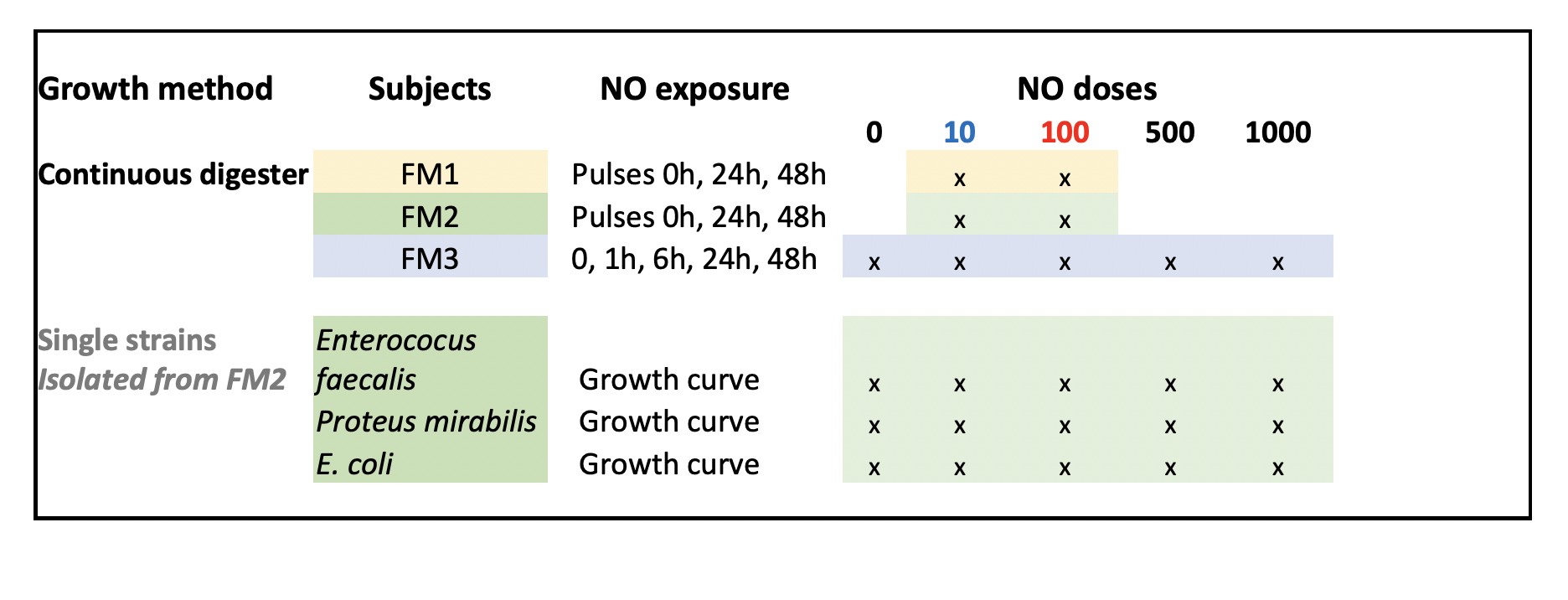

Supplement: FIG S1 [file msystems.00558-21-sf001.jpg]

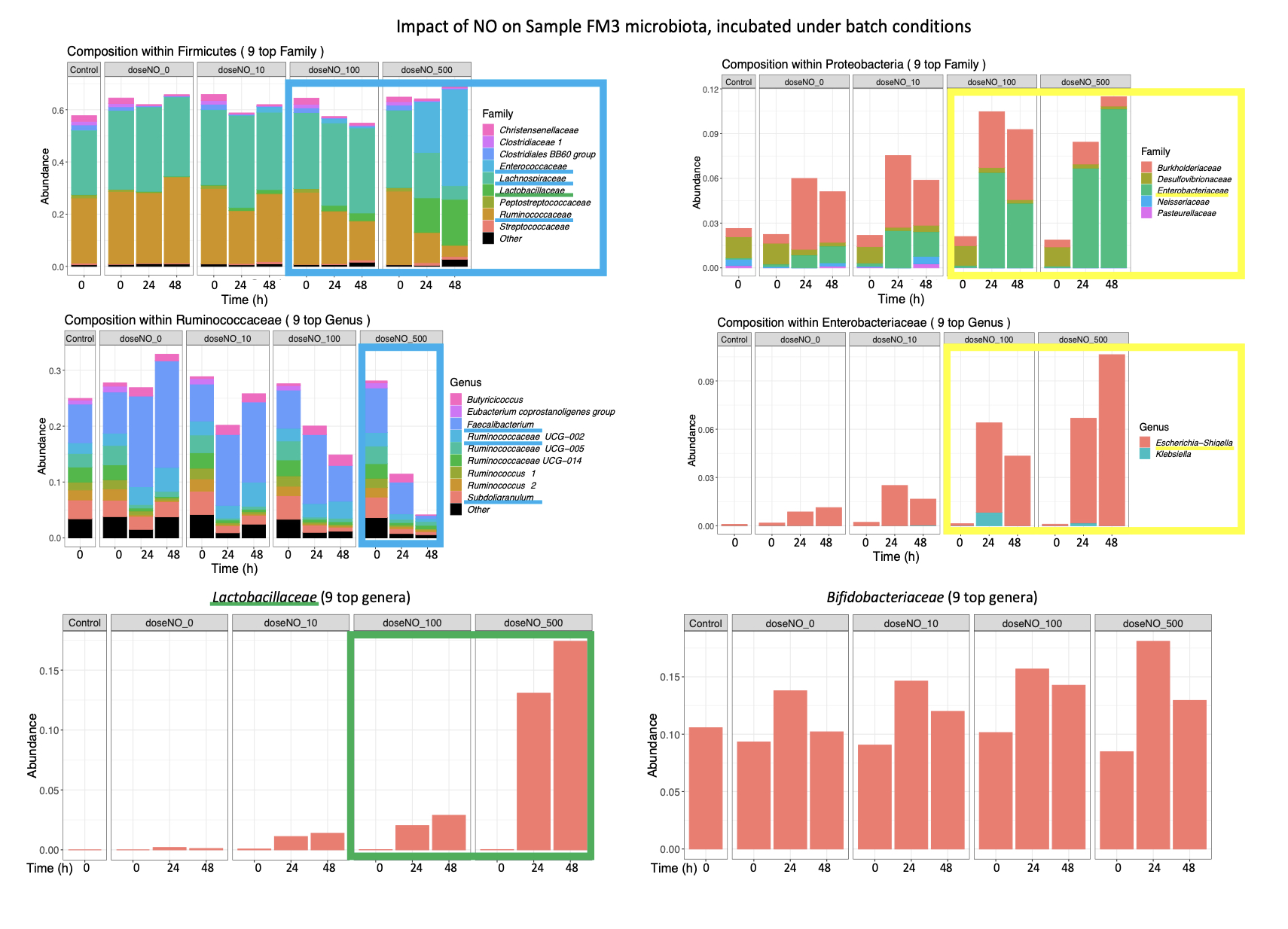

Supplement: FIG S2 [file msystems.00558-21-sf002.jpg]

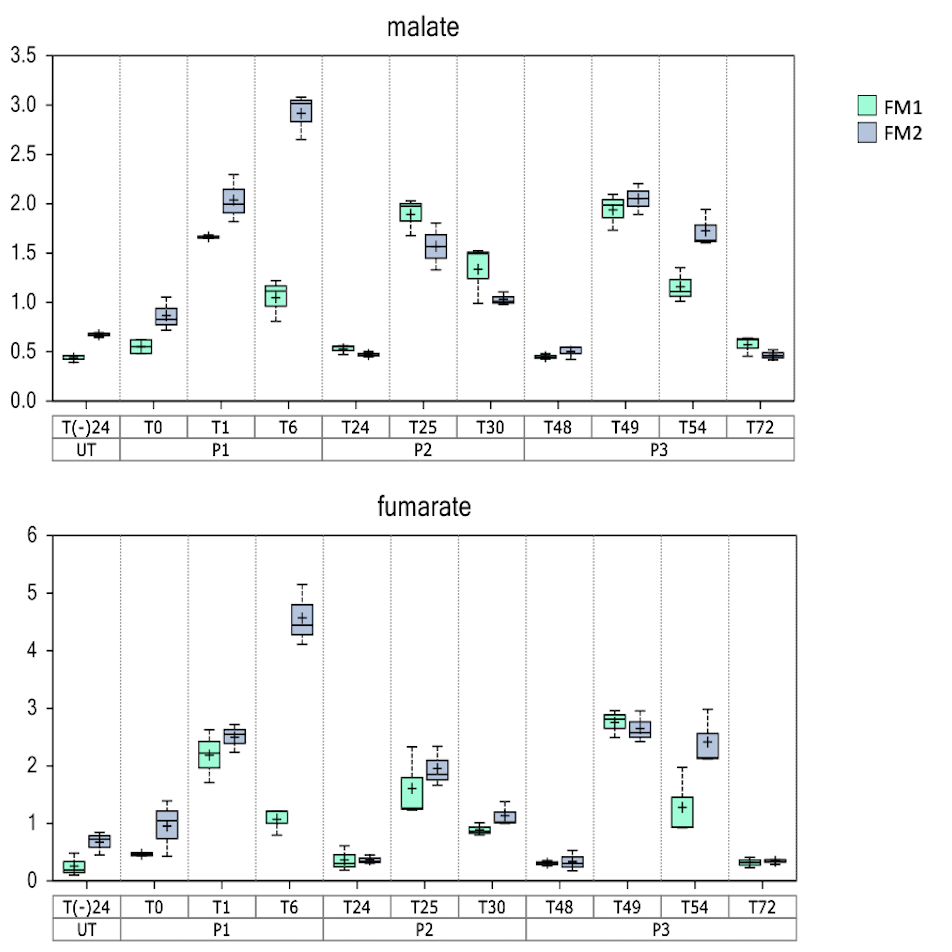

Supplement: FIG S3 [file msystems.00558-21-sf003.tif]
